# Domestic violence victimization among Chinese women and its relevance to their economic power

**DOI:** 10.3389/fsoc.2023.1178673

**Published:** 2023-04-17

**Authors:** Zixuan Wang, Takashi Sekiyama

**Affiliations:** Graduate School of Advanced Integrated Studies in Human Survivability, Kyoto University, Kyoto, Japan

**Keywords:** domestic violence, women, income level, influencing factors, China

## Abstract

**Introduction:**

This study conducted a survey of domestic violence victimization among women in China. Previously little research has been conducted on the subject of domestic violence against Chinese women as well as its relevance to their own economic power.

**Methods:**

Using online questionnaires, this study collected data about 412 women with current or previous marital status who came from four income brackets in Beijing and Shanghai.

**Results:**

It revealed that the proportions of physical, emotional, economic, and sexual violence they experienced were about 27.91%, 62.38%, 21.12%, and 30.10%, respectively. Women belonging to the highest income bracket faced almost the same risk of domestic violence compared with other income groups. Furthermore, there was a slight upward tendency in physical and emotional violence victimization in the highest-income group. The binary logistic regression analysis showed that adverse childhood experiences, arguments between couples due to different opinions regarding gender ideologies, and the approval level for specific gender ideologies were common significant factors across different income brackets. When all income brackets were considered, a higher income was tested as a protective factor with regard to sexual violence. As for the income gap between couples, women whose incomes were “once higher than that of the husband but now lower/almost the same” or “always higher than that of the husband” faced a higher risk of physical violence than women whose incomes were “always lower than/almost the same as that of the husband.”

**Discussion:**

This study not only revealed the reality of domestic violence victimization in China but also suggested that more attention should be paid to high-income women's domestic violence victimization as well as the importance of helping them both through academia and domestic violence support institutions.

## 1. Introduction

Intimate partner domestic violence, as a category of violence against women, causes serious physical and psychological damage to its victims (World Health Organization, 2005). Approximately 18% of the global population of women lives in China (The World Bank, 2021). Nevertheless, the issues of domestic violence against Chinese women as well as their own economic status have not been sufficiently unveiled in research. This presents a critical void with regard to clarifying the reality of worldwide domestic violence victimization.

In particular, the relationship between women's economic capacity and their domestic violence victimization has not been clearly answered in previous studies. Contradictory results have been reported on this question. Some literature and data have suggested that women with low economic power are more vulnerable to domestic violence (Dalal and Lindqvist, 2012, p. 271). The results of some economic support programs have also shown that increasing women's economic capacity can reduce their likelihood of experiencing intimate partner violence (Pronyk et al., 2006, p. 1981; Raghavendra et al., 2018, p. 16). Meanwhile, a few studies have implied that women who earn a higher income compared to that of their spouses may face a higher domestic violence risk (Abramsky et al., 2019, p. 11). Moreover, some studies conducted in Turkey show that women who were employed or had a personal income are more likely to experience intimate partner violence than women without employment or indivicual income (Alkan and Tekmanli, 2021, p. 12: Alkan and Ünver, 2021, p. 63).

The present study, therefore, aimed to investigate domestic violence victimization among Chinese women having different levels of personal income and its influencing factors. These specific research questions were formulated. How many percentages of Chinese women have experienced domestic violence? What is the distribution of domestic violence victimization rates among them with different incomes? Does the rate of domestic violence vary based on women's income? What factors contribute to experience of domestic violence among these women? To answer these questions, this study conducted an online questionnaire survey in China and analyzed the influencing factors with a binary logistic regression model.

## 2. Materials and methods

### 2.1. Literature review

#### 2.1.1. Definition and prevalence of domestic violence against women

In this article, the term domestic violence refers specifically to intimate partner violence, and it is divided into physical, emotional, economic, and sexual violence (Gender Equality Bureau Cabinet Office, 2021). Globally, 15–71% of women have reported experiencing physical or sexual violence perpetrated by an intimate partner at some point in their lifetime (World Health Organization, 2005). According to a national survey of women's social status that is conducted every decade in China, ~8.6% of women have experienced physical and emotional violence from their spouse (China Women's News, 2021). However, research investigating the victimization of Chinese women across different economic strata (e.g., based on personal income) has been scarce.

#### 2.1.2. The influencing factors for domestic violence against women

With regard to victimization, adverse childhood experiences (ACEs) (Whitfield et al., 2003, p. 176–178; Vung and Krantz, 2009, p. 710; Franklin and Kercher, 2012, p. 195), women's higher education level (Alkan et al., 2022, p. 12), the number of children (Alkan and Ünver, 2021, p. 63) and conservative attitudes and/or perceptions about traditional gender ideologies (Koenig et al., 2003, p. 285; Atkinson et al., 2005, p. 1145) have been identified as risk factors. Certainly, the problem of domestic violence cannot be explained by a single theory or factor. According to one ecological framework utilized by the World Health Organization, interpersonal violence may result from interactions among multiple factors at the individual, the relationship, the community, and the societal level. Personal experiences, biological factors, personal relationships, community circumstances in which social relationships occur, and societal factors influence whether violence is encouraged or inhibited are all said to be influencing factors with equal importance (World Health Organization, 2023).

#### 2.1.3. Economic power and domestic violence victimization

The relationship between economic power and domestic violence is complex. On the one hand, women's increasing/higher economic capacity has been treated as a protective element with regard to domestic violence. Low economic power and controlling behaviors from the husband have been identified as determinants of domestic violence (Dalal and Lindqvist, 2012, p. 271). Evidence has shown that decreases in the male-female wage gap may lead to a decrease in violence against women (Aizer, 2010, p. 1858). This mechanism can also be explained by the household bargaining theory, which implies that an increase in women's income will increase their bargaining power and thus reduce their likelihood of facing violence (Farmer and Tiefenthaler, 1997, p. 346). An intervention conducted in South Africa also revealed a drop in domestic violence victimization among women who received financial support (Pronyk et al., 2006, p. 1981). On the other hand, higher income may also place women at higher risk of facing severe violence and victimization. Studies have shown that wives who have jobs when their husbands do not or women who have higher incomes compared to that of their spouses are more likely to experience domestic violence (McCloskey, 1996, p. 458; Anderson, 1997, p. 667; Macmillan and Gartner, 1999, p. 957). Thus, it seems that women's tendency to blame men's inability to provide for them are significant motivators for such violence (Abramsky et al., 2019, p. 12).

### 2.2. Online questionnaire survey

The study data were obtained from questionnaires that were distributed and returned through a Chinese online questionnaire company; the study period stretched from August 25 to September 8, 2022. According to this company, ~10 million people answer questionnaires *via* its own platform. In order to ensure the sample source's reliability, only members who have passed the real name verification and keep active could be identified as valid (Wen, 2023). The major contents of the questionnaires used in this study referred to the Japanese Cabinet Office's questionnaire on domestic violence between spouses, and because of the simplicity of identifying the presence or absence of marital history, the subjects of this study were defined as women with a history of marriage. The procedure mainly included two steps: a screening survey and an official survey. In the screening survey, one questionnaire asked questions about gender (male or female) and intimate relationships (single/dating/married with no children/married with children/divorced/widowed) were used to build a sample frame for the official survey. With the limitation of funding, only two cities were included in the sample frame. Accordingly, women who lived in Beijing and Shanghai, which are two prosperous cities of China, and had current or previous marital status were selected as targets through the screening survey. Then simple random sampling was used to collect data from four income brackets. Every woman in the sample frame received the official survey questionnaire sent by the platform automatically in an equal manner. When the samples reached the required quantity, specialists of the company were responsible to exclude invalid answers. For example, to check if the user is answering the questions carefully, pop-up questions will appear on the screen while the questionnaire is being filled out. Moreover, a reasonable response time was also used to filter the answers.

One Chinese governmental data source (National Bureau of Statistics of China, 2021) showed that the average monthly income in Beijing and Shanghai were 11,199 and 10,500.5 CNY, respectively. Therefore, this study's observed income brackets were divided into four groups: almost no income (3,000 CNY per month), under average income (3,001–10,000 CNY per month), average income (10,001–20,000 CNY per month), and above average income (more than 20,000 CNY per month). The questionnaire contained items regarding experience of domestic violence and its influencing factors. Table 1 shows the main contents of the utilized questionnaire. For detailed information about the questionnaire, see the Supplementary material.

**Table 1:** Items included in the questionnaire.

**Question item**
1. The subject's age
2. The number of children
3. The subject's occupation
4. The subject's own income
6. Education level of subject's spouse/former spouse
7. Income gap and its changes between respondents and their spouses/former spouses
8. The ever experience of domestic violence
9. Treatments after experiencing violence
10. Adverse childhood experiences (ACEs)
11. Gender ideologies and related approval levels
12. Treatment related to arguments regarding differences in perceptions toward gender ideologies
13. Other unpleasant experiences in marital life

This study collected 412 valid samples from different income brackets in Beijing and Shanghai, China. Regarding the sample size determination, the data were collected from the four income groups as much as possible with the limited financial funding. Consequently, about 100 valid samples from each income group were collected.

### 2.3. Binary logistic regression analysis

This study adopted binary logistic regression analysis to reveal the factors influencing domestic violence victimization among Chinese women. The experiences of physical, emotional, economic, and sexual violence were set as the dependent variables. This study developed regression models for each of these four types of violence and ran the selected analysis for each income group. Referring to a similar survey conducted by the Japanese Cabinet Office (Gender Equality Bureau Cabinet Office, 2021) and literature related to the above-mentioned influencing factors, this study's independent variables included respondents' (1) number of children, (2) education level, (3) education level of spouse/former spouse, (4) income gap with spouse/former spouse, (5) history of ACEs, (6) degree of agreement with gender ideologies, (7) income level, and (8) frequency of arguments related to differences in gender ideologies. The assignment of variables is shown in Table 2.

**Table 2:** Assignment of variables.

**Variable**	**Assignment**
Education	Lower than university (including middle school, high school, vocational high school, and college) = 1, University = 0, Higher than university = 2 (including master's and doctoral)
Education of spouse/former spouse	Lower than university (including middle school, high school, vocational high school, and college) = 1, University = 0, Higher than university = 2 (including master's and doctoral)
Ever experience of domestic violence^a^	Yes = 1, No = 0
Income gap with spouse/former spouse	1 = Always lower than/about the same (within 10% difference) as that of the spouse/former spouse since the beginning of the relationship, 2 = Once lower than/about the same (within 10% difference) as that of the spouse/former spouse but now higher, 3 = Once higher than that of the spouse/former spouse but now lower /about the same (within 10% difference), 4 = Always higher than that of the spouse/former spouse since the beginning of the relationship, 5 = Have no idea
Adverse childhood experiences (ACEs)	1 = Never, 2 = Sometimes, 3 = Often, 4 = Frequently, 5 = Very frequently. In binary regression analysis, “never” and “sometimes” were coded as “0,” while “often,” “frequently,” and “very frequently” were coded as “1”
Arguments related to different gender ideologies	1 = Never, 2 = Sometimes, 3 = Often, 4 = Frequently, 5 = Very frequently. In binary regression analysis, “never” and “sometimes” were coded as “0,” while “often,” “frequently,” and “very frequently” were coded as “1”
Approval level toward gender ideologies	1 = Totally disagree, 2 = Disagree, 3 = Not sure, 4 = Agree, 5 = Completely agree

^a^Four types of violence were included: physical, emotional, economic, and sexual violence. Analysis of each type of violence was conducted separately. As long as the respondent selected “experienced” for one or more of the behavioral or descriptive options that belong to a type of violence in the questionnaire, she was considered to have experienced that violence and was coded as 1.

In the binary logistic regression model, a statistically significant influencing factor was defined as one that has an odds ratio (OR) value whose *p*-value is <0.05. An OR value >1 indicates a positive variation between independent and dependent variables, while an OR value smaller than one, indicates that the effect varies negatively. Furthermore, the OR value represents the level of influence of this independent variable. If the effect of an independent variable is significant, then a variable coded as 1 (e.g., “have children” and “yes, experienced”) is “OR” times more likely to eventually lead to the occurrence of the dependent variable than a variable coded as 0 (e.g., “no children” and “did not experience”). For example, an OR of 2 means that there is a 100% increase in the odds of an outcome with a given exposure. Alternatively, it could be illustrated that there is a doubling of the odds of the outcome. An OR of 0.2 means there is an 80% decrease in the odds of an outcome with a given exposure (Clay, 2018).

To determine the model's goodness of fit, this study used the Hosmer–Lemeshow test (H0 = Observed data and regression model fit well). A *p*-value exceeding the test level (*p* > 0.05) indicated that the information in the current data was sufficiently extracted, and the model fit was good (Zhou, 2020, p. 141–143).

## 3. Results

The basic information of the participants, including their education levels, education level of spouses/former spouses, income gap and its changes between couples, and number of children are presented in Table 3.

**Table 3:** Basic information of the respondents.

**Characteristics**	***n***	**%**
**Income group**		
Almost no income	103	25.00
Below average	102	24.76
Average	103	25.00
Above average	104	25.24
**Education** ^**a**^		
Junior high school	4	0.97
Vocational high school	4	0.97
High school	15	3.64
Junior college	59	14.32
University	264	64.08
Master's	64	15.53
Doctoral	1	0.24
Other	1	0.24
**Education** ^**b**^		
Junior high school	12	2.91
Vocational high school	6	1.46
High school	11	2.67
Junior college	71	17.23
University	231	56.07
Master's	72	17.48
Doctoral	9	2.18
Other	0	0
Have no idea	0	0
**Income gap and changes** ^**c**^		
Always lower/about the same^d^	239	58.01
Once lower/about the same but now higher	63	15.29
Once higher but now lower/about the same	89	21.60
Always higher	18	4.37
Have no idea	3	0.73
**Number of children**		
None	50	12.14
One or more	362	87.86

^a^Education level of respondents.

^b^Education level of respondents' spouse/former spouse.

^c^The comparison between respondents themselves and their spouse/former spouse.

^d^The difference was within 10%.

### 3.1. Domestic violence experiences across different income levels

**Figure 1:**
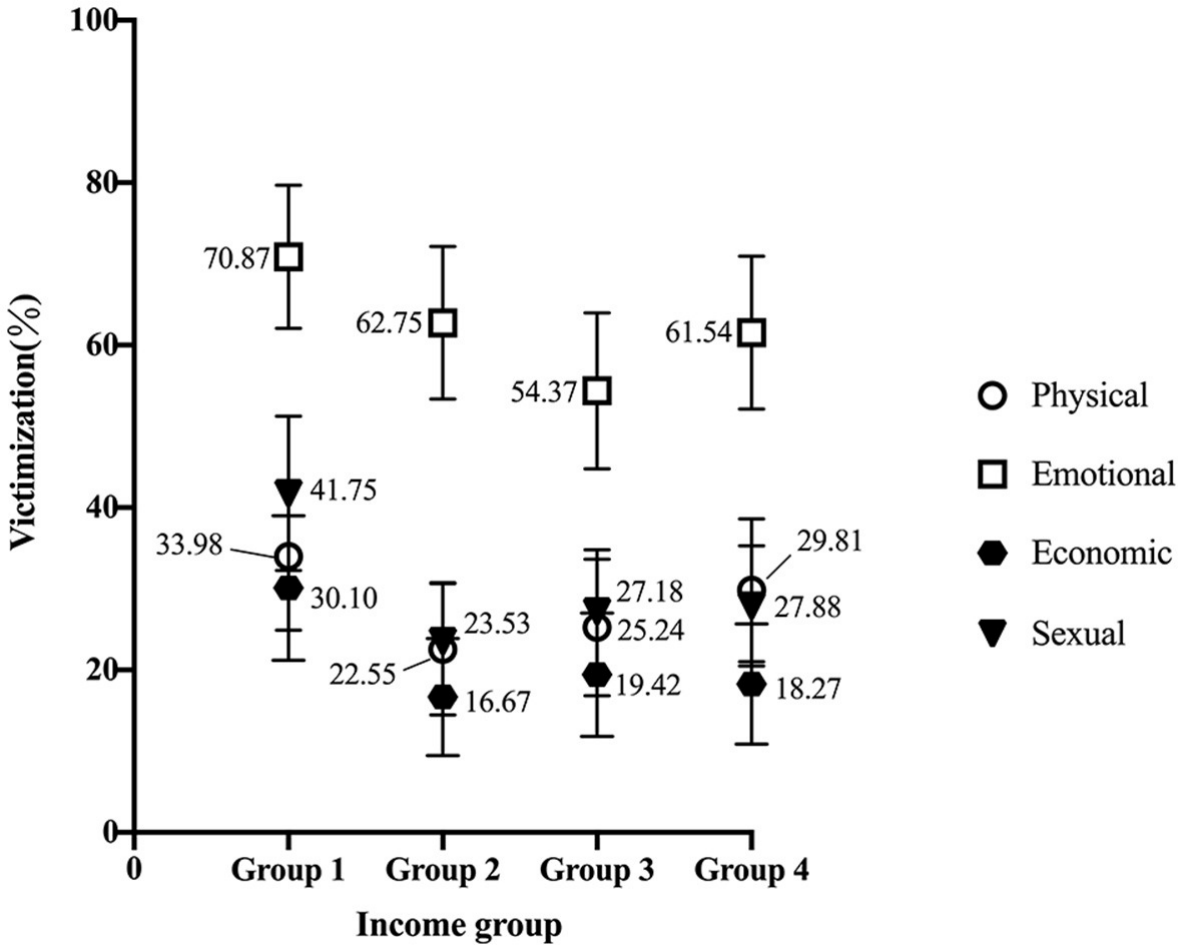
Results of domestic violence victimization.

Figure 1 presents analysis results regarding ever domestic violence victimization across the income groups. As the figure shows, victimization involving all types of violence was found to be highest in Income Group 1, which is the lowest income bracket. Furthermore, a slightly upward trend with regard to physical and emotional violence was observed in the highest income bracket (Income Group 4).

### 3.2. Gender ideologies

**Figure 2:**
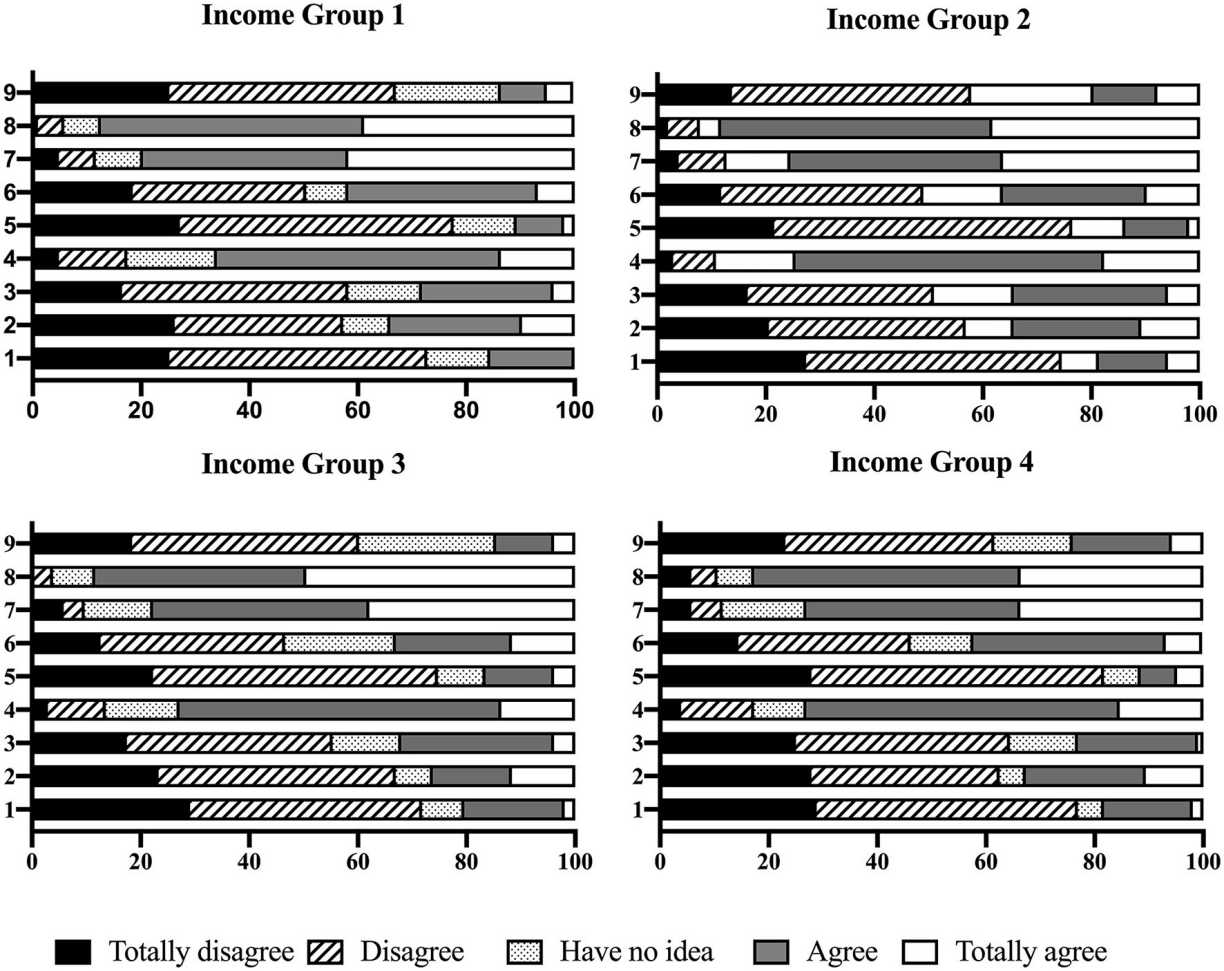
Agreement level of gender ideologies.

The analysis results for agreement levels regarding gender ideologies across the income groups are shown in Figure 2. Among the nine gender ideologies listed in the questionnaire, the following ideologies gained approval among respondents in all the income groups: “In public, the wife should take the husband's viewpoint as a priority and act accordingly,” “Being a housewife is a socially meaningful job,” and “Women should also focus on their working lives.” Although the levels were diverse, all the income groups showed a disapproving attitude toward the other ideologies in the questionnaire.

**Figure 3:**
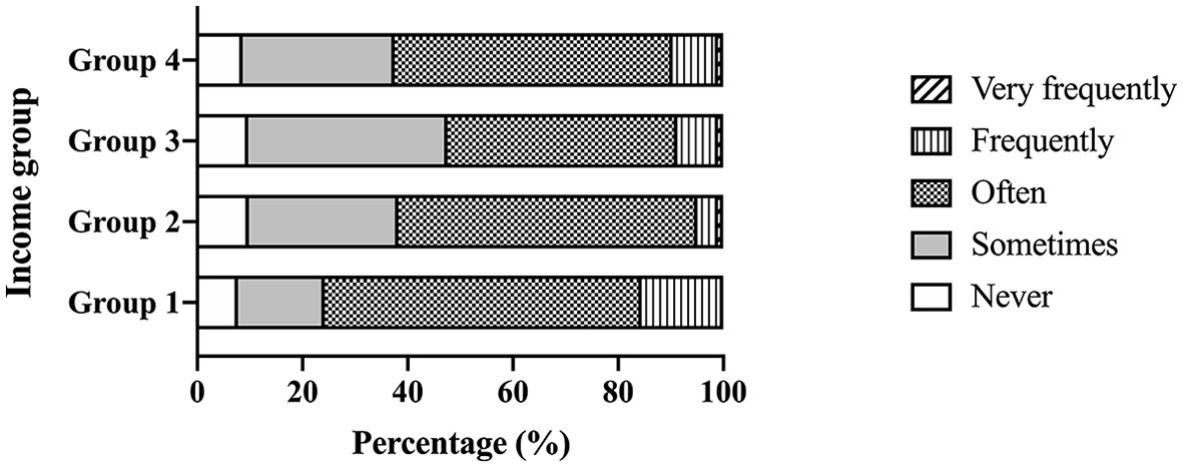
Frequency of arguments due to gender ideologies by income group.

Figure 3 presents the results of the frequency of arguments in relation to different perspectives regarding gender ideologies. As the figure shows, the frequency of arguments was highest in Income Group 1 and decreased sequentially in Income Groups 2 and 3. As the lowest frequency of arguments was observed in Income Group 3, the frequency of arguments increased in Income Group 4, which was lower than that in Income Group 1.

### 3.3. Adverse childhood experiences (ACEs)

**Figure 4:**
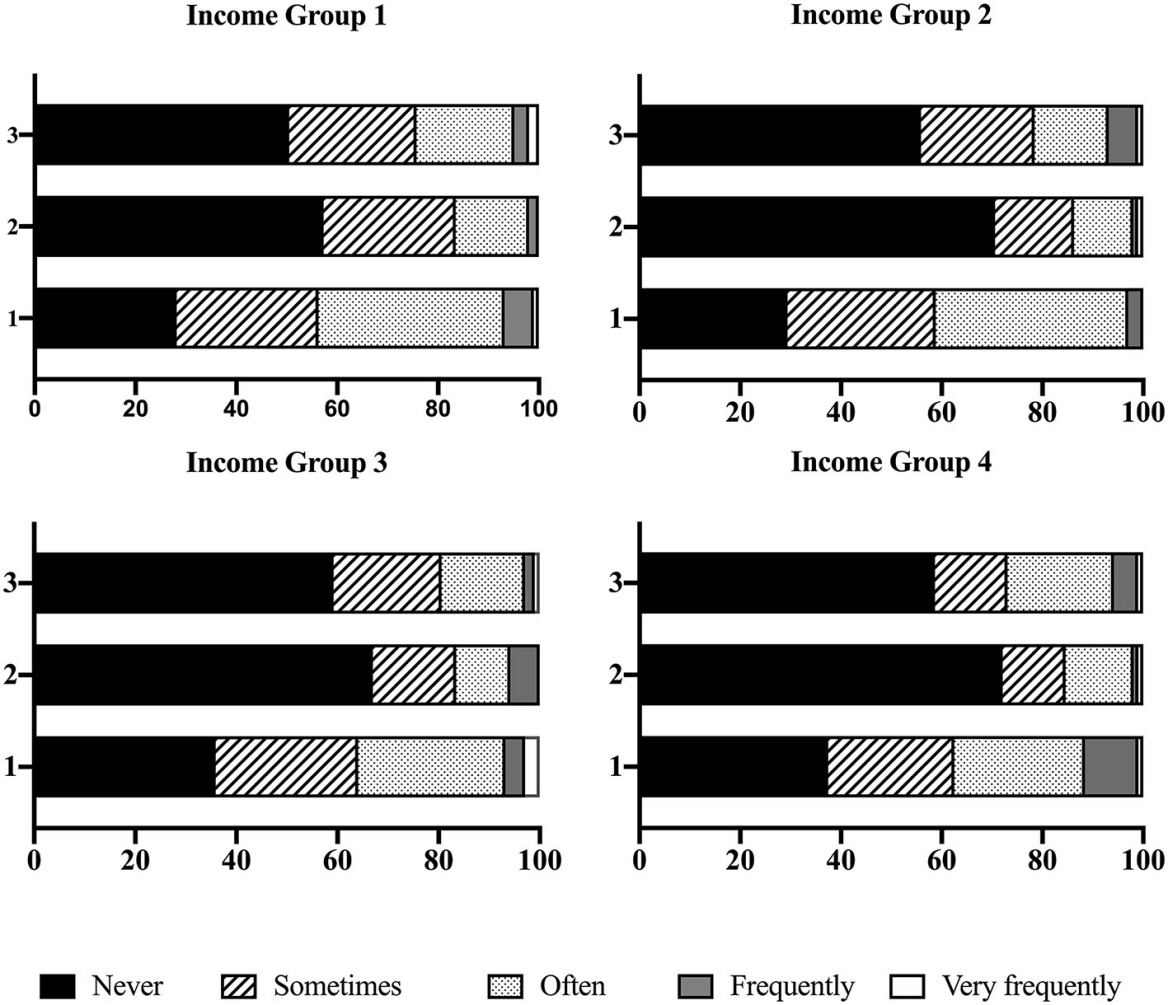
History of adverse childhood experiences (ACEs).

Figure 4 shows the history of ACEs in each income group. Income Group 1 had the highest rate of physical ACEs. The highest rates of experiencing sexual ACEs and witnessing ACEs were found in Income Groups 3 and 4, respectively.

### 3.4. Influencing factors

The results of the binary regression analysis for each income group are presented in Tables 4 through **8**.

**Table 4:** Binary regression analysis results of Group 1.

**Category**	**Significance**	**OR**	**95% C.I. for OR**	
			**LL**	**UL**
**Physical**				
ideoF	0.028	4.385	1.172	16.409
ideo^a^2	0.008	1.715	1.154	2.549
**Emotional**				
ideoF	0.033	3.344	1.102	10.144
ideo2	0.024	1.634	1.067	2.503
ideo6	0.048	1.510	1.003	2.272
**Economic**				
ACEs	0.002	6.566	1.958	22.015
ideo1	0.042	1.861	1.023	3.384
ideo3	0.007	1.921	1.195	3.087
ideo8	0.020	2.539	1.156	5.576
**Sexual**				
ACEp	0.017	2.975	1.217	7.271
ideo8	0.019	2.200	1.138	4.254

OR, odds ratio; CI, confidence interval; LL, lower limit; UL, upper limit; ideoF, quarrels owing to differences in perceptions of gender ideologies.

^a^Approval level of gender ideologies: 1 = Husbands should work outside the home, and wives should protect the home; 2 = It is the man's responsibility to provide for and protect his family; 3 = Women do not have to work if they are not economically disadvantaged; 6 = Women are better suited for housework and childcare than men; 8 = Women should also focus on their professional lives. ACEs = Experience of sexual violence from older family members during childhood. ACEp = Experience of physical violence from older family members during childhood.

#### 3.4.1. Differences in income levels

In the income group with “almost no income,” there were two major risk factors for physical violence, three for emotional violence, four for economic violence, and two for sexual violence victimization (Table 4). Among these, approving attitudes toward certain gender ideologies were found to be risk factors for every type of domestic violence. Regarding the group that had “below the average” incomes, one risk factor for economic violence and two factors each for the other types of domestic violence were observed (Table 5). Furthermore, this is the only income group where ACEs did not wield any significant influence. For the group with “average level” incomes, three risk factors for physical violence, four for emotional violence, one for economic violence, and three for sexual violence were considered significant (Table 6). ACEs formed the most frequently observed risk factor. For the group with “above average” incomes, the number of influencing factors for physical, emotional, economic, and sexual violence were three, three, two, and three, respectively (Table 7).

**Table 5:** Binary regression analysis result of Group 2.

**Category**	**Significance**	**OR**	**95% C.I. for OR**	
			**LL**	**UL**
**Physical**				
ideoF	0.008	7.919	1.696	36.981
ideo^**a**^9	0.028	0.469	0.239	0.921
**Emotional**				
ideoF	<0.001	5.670	2.243	14.334
ideo2	0.025	1.690	1.068	2.676
**Economic**				
ideoF	0.037	5.278	1.109	25.127
**Sexual**				
ideoF	0.028	3.787	1.151	12.459
ideo7	0.038	0.611	0.383	0.972

OR, odds ratio; CI, confidence interval; LL, lower limit; UL, upper limit; ideoF, quarrels owing to differences in perceptions of gender ideologies.

^a^Approval level of gender ideologies: 2 = It is the man's responsibility to provide for and protect his family; 7 = Being a housewife is a socially meaningful job; 9 = Housewives are better off in many ways than women who work outside the home.

**Table 6:** Binary regression analysis result of Group 3.

**Category**	**Significance**	**OR**	**95% C.I. for OR**	
			**LL**	**UL**
**Physical**				
ACEs	0.013	5.011	1.399	17.947
ACEm	0.019	5.272	1.320	21.058
ideo^a^1	0.015	1.959	1.137	3.377
**Emotional**				
ACEm	0.035	6.667	1.144	38.862
ideoF	0.003	4.137	1.646	10.398
ideo5	0.040	0.570	0.334	0.975
ideo7	0.010	1.786	1.147	2.783
**Economic**				
ACEs	0.042	3.579	1.045	12.260
**Sexual**				
ACEm	0.040	3.877	1.061	14.164
ideoF	0.017	3.699	1.263	10.833
ideo5	0.027	0.478	0.248	0.921

OR, odds ratio; CI, confidence interval; LL, lower limit; UL, upper limit; ideoF, quarrels owing to differences in perceptions of gender ideologies.

^a^Approval level of gender ideologies: 1 = Husbands should work outside the home, and wives should protect the home; 5 = Husbands should decide important matters in marriage; 7 = Being a housewife is a socially meaningful job. ACEm: Experience of witnessing violence against mother/stepmother from family members during childhood. ACEs: Experience of sexual violence from older family members during childhood.

**Table 7:** Binary regression analysis result of Group 4.

**Category**	**Significance**	**OR**	**95% C.I. for OR**	
			**LL**	**UL**
**Physical**				
ACEs	0.029	3.798	1.145	12.593
ideo^a^4	0.037	0.558	0.322	0.967
ideo5	0.003	2.621	1.38	4.977
**Emotional**				
ideoF	0.047	2.436	1.013	5.857
ideo7	0.014	1.775	1.124	2.805
ideo9	0.041	0.584	0.349	0.977
**Economic**				
ACEs	0.023	4.485	1.226	16.407
ideoF	0.024	6.223	1.276	30.363
**Sexual**				
ACEs	0.008	5.269	1.556	17.847
ideo6	0.028	1.720	1.059	2.794
ideo7	0.012	2.061	1.169	3.635

OR, odds ratio; CI, confidence interval; LL, lower limit; UL, upper limit; ideoF, quarrels owing to differences in perceptions of gender ideologies.

^a^Approval level of gender ideologies: 4 = In public, the wife should take the husband's viewpoint as a priority and act accordingly; 5 = Husbands should decide important matters in marriage; 6 = Women are better suited for housework and childcare than men; 7 = Being a housewife is a socially meaningful job; 9 = Housewives are better off in many ways than women who work outside the home. ACEs: Experience of sexual violence from older family members during childhood.

#### 3.4.2. Arguments regarding gender ideologies

Arguments that stemmed from different perspectives regarding gender ideologies were the most common risk factor across all the income groups. As shown in Tables 4–7, women who frequently argued with their spouses/former spouses were more likely to experience domestic violence than those who did not argue or rarely argued with their spouses/former spouses. Specifically, the former were 143.6–691.9% more likely to experience intimate partner violence.

#### 3.4.3. Approval levels for specific gender ideologies

In this survey, the favorable/unfavorable tendencies for each of the nine gender ideologies did not differ significantly across the four income groups. Overall, approving attitudes toward traditional gender ideologies (e.g., “The man is responsible for providing and protecting his family,” “A husband should take a job outside of the home, and a wife should take care of the home,” “Women do not have to work if they are not economically disadvantaged,” etc.) was risky for subjects participating in this survey, especially for Income Group 1, where the number of traditional gender ideologies that wielded a significant influence was more than those in the other income groups. Nevertheless, different scenarios were observed. First, support for certain gender ideologies was found to be a protective factor against domestic violence among the study participants. As Table 8 shows, when agreement levels regarding the gender ideology that “husbands should decide important matters in marriage” increased by one unit, women in Income Group 3 were found to be 43.0 and 52.2% less likely to experience emotional and sexual violence from their spouses, respectively. With every unit increase in the degree of agreement with the gender ideology that “housewives are better off in many ways than women who work outside the home”, women in Income Group 2 (Table 5) were found to be 53.1% less likely to experience physical violence, and women belonging to Income Group 4 (Table 7) were found to be 41.6% less likely to suffer emotional violence from their spouses. Furthermore, support levels for the same idea brought about opposite effects on victimization in different groups of women. On the one hand, in Income Group 2, as the preference for the gender ideology that “being a housewife is a socially meaningful job” increased by each unit, women in that group were found to experience a 38.9% decrease in the likelihood of experiencing sexual violence. On the other hand, in income groups 3 and 4, for every unit increase in approving attitude toward this idea, the possibility of women experiencing emotional violence was found to increase by 78.6 and 77.5%, respectively. Furthermore, women belonging to Income Group 4 were 106.1% more likely to experience sexual violence.

**Table 8:** Binary regression analysis result: all income groups considered.

**Category**	**Significance**	**OR**	**95% C.I. for OR**	
			**LL**	**UL**
**Physical**				
ideoF	0.028	4.385	1.172	16.409
ideo^a^2	0.034	1.238	1.016	1.508
Incomegap^b^(2)	0.010	2.020	1.182	3.453
Incomegap(3)	0.008	3.780	1.426	10.022
**Emotional**				
ideoF	0.033	3.344	1.102	10.144
ideo2	0.004	1.335	1.098	1.622
ideo3	0.025	1.265	1.029	1.556
ideo7	0.011	1.293	1.062	1.576
**Economic**				
ACEs	0.002	6.566	1.958	22.015
**Sexual**				
ACEp	0.017	2.975	1.217	7.271
ideo2	0.017	1.260	1.042	1.522
income^c^(1)	0.006	0.429	0.235	0.784
income(2)	0.029	0.521	0.290	0.935
income(3)	0.037	0.540	0.302	0.964

OR, odds ratio; CI, confidence interval; LL, lower limit; UL, upper limit; ideoF, quarrels owing to differences in perceptions of gender ideologies.

^a^Approval level of gender ideologies: 2 = It is the man's responsibility to provide for and protect his family; 3 = Women do not have to work if they are not economically disadvantaged; 7 = Being a housewife is a socially meaningful job.

^b^Income gap and its changes between respondents and their spouses/former spouses (all compared with “always lower/about the same”): (2) = once higher than husband but now lower/almost the same; (3) = always higher than husband.

^c^Income group [all compared with income Group 1 (“almost no income”)]: (1) = below average; (2) = average; (3) = above average. ACEp: Experience of physical violence from older family members during childhood. ACEs: Experience of sexual violence from older family members during childhood.

#### 3.4.4. Women's income

As Table 8 shows, when all the income brackets data were inputted into the model, income level was found to be a significant factor with regard to sexual violence. Compared to women belonging to the group with “almost no income,” women with “below-average” income were 57.1% less likely to experience sexual violence. The groups with “average level” incomes and “above-average” incomes were 47.9 and 46.0%, respectively, less likely to experience sexual violence.

#### 3.4.5. Income gaps within couples

When all of the income bracket data were combined, the income gaps within couples presented a significant influence on physical violence victimization (see Table 8). Women whose incomes were “once higher than that of the husband but now lower/almost the same” and “always higher than that of the husband” were found to face a higher risk of physical violence than women whose incomes were “always lower than/almost the same as that of the husband.” Specifically, women with incomes that were “once higher than that of the husband but now lower/almost the same” were 102.0% more likely to experience physical violence from their spouses. For women with incomes that were “always higher than that of the husband,” this possibility was 278.0%.

## 4. Discussion

### 4.1. Prevalence of domestic violence against Chinese women

This survey found that about 27.91, 62.38, 21.12, and 30.15% of women experienced physical, emotional, economic, and sexual violence, respectively, from their spouses/former spouses; these figures are much higher than the data reported thus far (China Women's News, 2021). This suggests that such cases may be under-reported in Chinese society.

### 4.2. Domestic violence experiences across different income levels

Domestic violence victimization rates among the four income groups were close (see Figure 1), even between the lowest and highest income groups. Furthermore, a slight upward tendency regarding physical and emotional violence victimization was observed in the highest-income group. Overall, it is hard to say that women with higher incomes are safer from domestic violence than lower-income women.

### 4.3. Influencing factors

Although the vast majority of references about influencing factors were drawn from the literature of Western societies, the findings suggest a commonality between experiences of domestic violence among women from both Eastern and Western sociocultural backgrounds and contexts. Binary logistic regression analysis showed that ACEs, arguments between couples because of their different opinions regarding gender ideologies, and approval levels toward specific gender ideologies were common significant factors for domestic violence across all income brackets. When each income group was examined separately, most of the risk factors identified in past research on domestic violence in Western societies were found to have a significant impact.

When all income brackets were considered, the income levels of women and income gaps within couples were found to be significant factors. This study found that a higher personal income level functioned as a protective safeguard against sexual domestic violence for respondents though it had no significant influence on the other types of violence. Contrary to this finding, some studies mentioned that women who were employed were prone to be exposed to sexual violence compared with women who have no job (Alkan and Tekmanli, 2021, p.12).

Furthermore, respondents whose income was higher than that of their husbands were more likely to experience physical domestic violence than respondents whose income was lower than/nearly the same as that of spouses/former spouses. This result is partly consistent with some implications from previous studies. One study found that narrowing the wage gap between men and women led to a reduction in domestic violence rates (Aizer, 2010, p. 1847). On the other hand, another study found that women who earned more than their partners could face greater risks of physical and sexual intimate partner violence (Abramsky et al., 2019, p. 11). Thus, this current study's result implies a worrying sign that, as an increasing number of women begin to participate in the workplace in Chinese society and the economic status of women continues to rise, the risk of domestic violence may also follow an upward trend within the family.

In addition to financial ability, ACEs were linked to future experiences of domestic violence in adulthood. Similar results were found in some previous studies (Whitfield et al., 2003, p.176–178; Vung and Krantz, 2009, p. 710; Franklin and Kercher, 2012, p. 195). This revealed the importance of providing healthy growing-up environments for girls; this will allow them to build healthier marriages in adulthood.

Furthermore, arguments within couples with regard to different perspectives on traditional gender ideologies as well as women's attitudes toward these concepts were both confirmed to be influencing factors. Previous studies have indicated that men's opinions regarding gender ideologies (Atkinson et al., 2005, p. 1145) and tensions within couples (Abramsky et al., 2019, p. 11) were factors influencing women's domestic violence victimization. This study investigated women's attitudes toward gender ideologies as well as their arguments with spouses/former spouses. It found that arguments regarding gender ideologies formed a domestic violence risk factor for the respondents, regardless of their income level. On the other hand, a higher level of agreement with traditional gender ideologies could place women in unsafe situations; this was especially the case for those who belonged to the lowest income group (“almost no income”). Among them, a higher agreement level toward gender ideologies that valued the voice of men or the role of housewives in the family led to increased domestic violence risk factors. Furthermore, approving attitudes toward specific ideologies may create different influences on risk of domestic violence among different income groups.

This study had some limitations that must be considered. First, because the questionnaire survey was conducted online, this study could not determine whether the respondents gave false answers. Furthermore, those who could not or did not use the Internet and those who could not or did not access the data platform of the study's selected web survey company were practically excluded from the reach of its questionnaire. Second, certain financial constraints prevented the full development of the items and sample size of the current questionnaire. Thus, a more reliable interpretation will require a larger-scale survey. Finally, the main theoretical basis of this study was drawn from prior research in non-East Asian countries/regions, and this may result in inadequate explanations regarding the context of Asian culture.

## 5. Conclusion

Previously little research has been conducted on the subject of domestic violence against Chinese women as well as its relevance to their own economic power. Also, in a global context, previous studies reported contradictory results on the relationship between women's economic capacity and their domestic violence victimization. Namely, some literature suggested that women with low income are more vulnerable to domestic violence while a few studies implied that women with high income face a higher domestic violence risk.

This study, therefore, investigated domestic violence victimization and its influencing factors among women across different income groups in China. Data from 412 women belonging to four income brackets in two cities (Beijing and Shanghai) were collected through online questionnaires. This study adopted a logistic regression model to analyze the factors influencing domestic violence victimization across these groups.

It can be said that this study makes a significant contribution to the literature because it revealed that domestic violence victimization rates among Chinese women who were from multiple economic strata of society and who had multiple earning capacities. The primary results showed that about 27.91 and 62.38% of Chinese women who participated in this study had experienced physical and emotional domestic violence, respectively, which is much higher than the results obtained by the Chinese government. This difference may come from the different definitions and descriptions of each type of violence used in this study and the Chinese government's. Or perhaps the inherent nature of on-site surveys with interviewers, which was adopted by the Chinese government's survey, and online questionnaires that rely on subjects' understanding resulted in the difference. This study also revealed that women with higher incomes faced almost the same risk of victimization compared to low-income women. Furthermore, binary logistic regression model analysis revealed that ACEs, frequent arguments regarding different perceptions of gender ideologies within couples, and approval levels regarding gender ideologies had significant effects on domestic violence victimization among women regardless of income level or type of violence. Regarding physical violence, women who earned/once earned more than their spouse/former spouse faced greater risks than those who earned lower than/almost the same income as that of their partners. Regarding sexual violence victimization, a higher income seemed to have negative effects. The current study findings also suggested that women with higher income could face a similar risk of domestic violence, although the victimization of low-income women has received much more attention. Accordingly, the needs of women with high economic capacities must be investigated and considered while formulating and implementing domestic violence support policies.

Future research must aim to not only overcome the limitations listed above but also to resolve the questions unveiled in this study. For instance, enlarging the sample size of each income bracket is important for drawing reliable conclusions. Furthermore, the specific mechanisms operating between domestic violence victimization and the observed influencing factors must be examined. Finally, differences in the factors underpinning domestic violence victimization in different countries or regions must also be revealed.

## Data availability statement

The raw data supporting the conclusions of this article will be made available by the authors, without undue reservation.

## Ethics statement

Ethical review and approval was not required for the study on human participants in accordance with the local legislation and institutional requirements. The patients/participants provided their written informed consent to participate in this study.

## Author contributions

ZW: original draft, methodology, data collection, analysis, statistics, software, and draft review. TS: methodology, supervision, and review of the draft. All authors contributed to the article and approved the submitted version.

## Supplementary material

Supplementary material 1 The questionnaire contents.
